# Pre- and postnatal exposure to legacy environmental contaminants and sensation seeking in Inuit adolescents from Nunavik

**DOI:** 10.1371/journal.pgph.0002478

**Published:** 2023-10-18

**Authors:** Avril Gagnon-Chauvin, Sandra W. Jacobson, Joseph L. Jacobson, Mathieu Fornasier-Bélanger, Yohann Courtemanche, Pierre Ayotte, Richard E. Bélanger, Gina Muckle, Dave Saint-Amour

**Affiliations:** 1 Département de Psychologie, Université du Québec à Montréal, Montréal (Québec), Canada; 2 Centre de Recherche du CHU Sainte-Justine, Montréal (Québec), Canada; 3 Department of Psychiatry and Behavioral Neurosciences, Wayne State University School of Medicine, Detroit, Michigan, United States of America; 4 Centre de Recherche du CHU de Québec-Université Laval, Québec (Québec), Canada; 5 Département de Médecine Sociale et Préventive, Faculté de Médecine, Pavillon Ferdinand-Vandry, Université Laval, Québec (Québec), Canada; 6 Département de Pédiatrie, Université Laval, Centre mère-enfant Soleil du CHU de Québec, Québec (Québec), Canada; 7 École de Psychologie, Université Laval, Québec (Québec), Canada; University of Ghana School of Public Health, GHANA

## Abstract

Despite extensive evidence from cohort studies linking exposure to lead (Pb), mercury (Hg) and polychlorinated biphenyls (PCBs) to numerous cognitive outcomes in children and adolescents, very few studies addressed reward sensitivity, a key dimension of emotional regulation. The present study aimed to examine associations between pre- and postnatal exposure to these environmental neurotoxicants and sensation seeking, a behavioral feature of reward. A total of 207 Inuit adolescents (mean age = 18.5, SD = 1.2) from Nunavik, Canada, completed the Brief Sensation Seeking Scale (BSSS-4) and Sensation Seeking– 2 (SS-2), two self-report questionnaires assessing proneness to sensation seeking. Prenatal, childhood and adolescent exposure to Pb, Hg and PCBs were measured in cord blood at birth and blood samples at 11 years of age and at time of testing. Multiple linear regression models were performed, potential confounders including participants’ sociodemographic characteristics and nutrient fish intake were considered. Results showed that higher child blood levels of Pb (b = -0.18, *p* = 0.01) and PCB-153 (b = -0.16, *p* = 0.06) were associated with lower BSSS-4 total scores, while cord and adolescent blood PCB-153 levels were significantly related to lower SS2 total scores (b = -0.15, *p* = 0.04; b = -0.24, *p* = 0.004). Such associations persisted after further adjustment for co-exposure to concurrent contaminants. These associations were influenced by self-report positive affect and marginally moderated by sex. Sex differences were only observed for child PCB exposure, with the association for risk-taking sensation seeking observed only in girls but not in boys. Further research is warranted to assess the extent to which reduced sensation seeking in chronically exposed individuals affects their behaviors, well-being, and emotional regulation.

## Introduction

Inuit communities from Nunavik, a region located in Northern Quebec, Canada, are particularly exposed to legacy (i.e., recognized) environmental contaminants, such as lead (Pb), mercury (Hg) and polychlorinated biphenyls (PCBs). In fact, long-range atmospheric transport, waterways, and oceanic currents are known to transport large amounts of toxic compounds from industrialized countries to the Arctic, including Nunavik coastal regions [1, 2]. Inuit populations from Nunavik show significantly higher exposure levels than general Canadian population [3], as they traditionally base their diet on hunting and fishing, including consumption of large fish and marine mammals (e.g., Arctic char, beluga) [4].

People in Nunavik appear at greater risk to experience so-called risky behaviors, such as psychoactive substance uses, including cigarette smoking, episodic excessive alcohol consumption, and cannabis use, as well as gambling [5, 6]. Various factors such as low socioeconomic status (SES), colonial policies, decreased opportunities, lack of psychosocial health services, victimization and acculturation, can contribute to the high prevalence of such risky behaviors in Inuit communities [7]. However, the potential contribution of neurotoxicant exposure to these behaviors has yet to be studied. Despite numerous large cohort studies showing alterations of cognitive function with prenatal exposure to legacy contaminants, limited attention has been directed towards emotional and motivational outcomes [8]. While several studies have shown higher rates of depression and anxiety symptoms as well as a lack of positive affect in various cohorts of chronically-exposed individuals [9–13], very few have examined reward processes. Reward dysregulation is thought to play a key role in various psychopathologies (e.g., depression, addiction, substance abuse, attention and deficit disorder) and risk-taking behaviors [14] and is a central aspect of hedonic experiences, motivation towards goal-oriented actions, reinforcement learning and reward-related decision making [15].

Our current knowledge of how neurotoxicants can alter reward processing comes from animal studies. Animals exposed to Pb postnatally showed higher response rates in fixed-interval [16, 17] and fixed-ratio schedule paradigms [18], suggesting an inability to inhibit inappropriate responses toward reinforcement. Experimental models have also repeatedly shown that higher gestational Hg exposure was associated with motor impulsivity in operant tasks [19–21]. Similarly, animals both prenatally and/or postnatally exposed to PCBs presented shorter inter-response times (IRTs) in differential reinforcement of low-rate (DRL) paradigms and in fixed-interval reinforcement schedules [22–24], pointing to a marked motor impulsivity during reward-related tasks in exposed subjects. In sum, these studies suggest an enhanced sensitivity to reinforcement and deficits in inhibitory control towards reinforcement following exposure to Pb, Hg and PCBs.

Only one study assessing neurotoxicant effects specifically on behavioral reward processing has been conducted in humans [25]. Stewart et al. examined reward behaviors in 167 children at 9.5 years of age who were exposed to Pb, Hg and PCBs, using a differential reinforcement of low rates (DRL) 20-second fixed-interval schedule. In this task, the ongoing interval was reset, and no reinforcement (5 cents worth of marbles) was delivered if the participant responded before the end of the interval delay, thus reducing the total amount of money earned at the end of the task. In line with experimental studies, Stewart et al. [25] observed that postnatal exposure to Pb, as well as prenatal exposure to organic Hg and PCBs, were significantly associated with excessive responding, lower inter-response times and fewer reinforced responses across the task, suggesting impaired inhibition control and difficulty delaying rewards as a result of chronic contaminant exposure.

Self-report measures of reward-related behaviors is complementary to behavioral tasks and may be more appropriate for studying large human cohorts. Among these measures, self-reported sensation seeking is a widely used behavioral feature allowing reward processing assessment. Sensation seeking can be defined as the search for novelty, sensations and experiences that are varied, intense and complex, and as the propensity to engage in risky physical, social, legal and financial behaviors to attain such experiences [26]. Compared to low sensation-seekers, high sensation-seekers tend to be more sensitive to reward, while presenting greater tolerance to loss [27–29]. Therefore, they tend to engage in more risky behaviors (e.g., substance use and abuse, reckless driving, risky sexual activities, gambling) that provide intense and often immediate rewarding sensations or experiences [30, 31].

Throughout development, sensation seeking is thought to follow a curvilinear trend, reaching its peak in the middle of adolescence and stabilizing in early adulthood [32, 33]. Across all age groups, males tend to present heightened sensation seeking compared to females [34]. Such temporal and sex-related trends correlate with the well-established high rates of risk-taking behaviors during adolescence [35, 36]. Moreover, affective traits and states as well as mood impairments have been shown to be associated with sensation seeking [37–39], pointing out the importance of taking into account emotional states when studying reward processes, particularly in adolescence, which is a critical developmental period during which health-related behaviors are likely to establish and persist through adulthood [35, 40].

In the present study, our aim was to investigate whether Pb, Hg or PCBs measured at birth, childhood and adolescence are associated with sensation seeking in Inuit adolescents. Based on previous studies showing associations between reward sensitivity and Pb, Hg and PCB exposures, we hypothesized that higher blood contaminant levels will be associated with higher sensation seeking scores. We also explored the potential influence of affective functioning, as well as potential sex moderation effects, on associations between contaminant exposure and sensation seeking.

## Materials and methods

### Study setting and population

**Fig 1:**
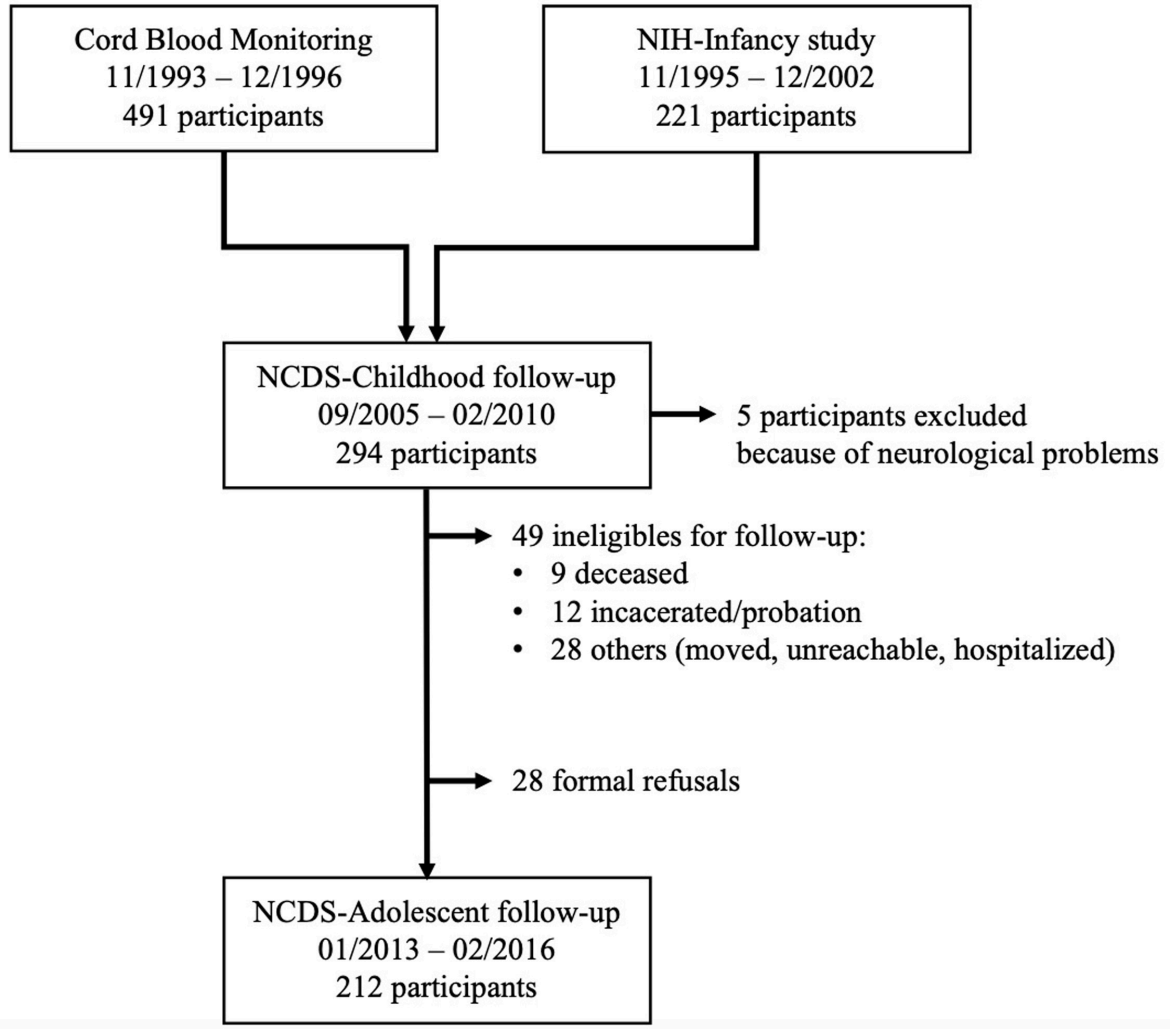
Flow chart for recruitment and follow-up of study participants from November 1993 to February 2016. NIH, National Institutes of Health; NCDS, Nunavik Child Development Study.

The present study is part of a larger adolescent follow-up of the Nunavik Child Development Study (NCDS), originally conducted among the Nunavik Cord Blood Monitoring Program and the National Institutes of Health (NIH) prospective infancy study [1, 41]. As illustrated in Fig 1, pregnant Inuit women from Nunavik were initially recruited between 1993 and 1998 in the Cord Blood Monitoring Program (*N* = 491) [42] and between 1995 and 2002 in the NIH prospective infancy study (*N* = 221) [1] to document prenatal exposure to environmental contaminants in Nunavik, using an observational cohort design. An NCDS follow-up study (*N* = 294) was conducted among these Inuit families (NCDS-Childhood) to examine associations between pre- and postnatal exposures to environmental contaminants and neurobehavioral outcomes in children at 11 years of age [43]. Recruitment methodologies for the Cord Blood Monitoring Program, the NIH Prospective Infancy Study and the NCDS-Childhood are described elsewhere [1, 44, 45].

Between January 2013 and February 2016, NCDS-Childhood participants were examined again as part of an adolescent follow-up study. Inclusion criteria for the NCDS-Adolescent follow-up included being a resident of Nunavik and, for cost-efficiency reasons, being able to meet with the research team in one of the three largest Nunavik villages for assessment. During recruitment, 5 adolescents were excluded due to severe health or neurological problems unrelated to exposure at the time of the NCDS-Childhood interview (epilepsy *n* = 2; head trauma *n* = 1, meningitis *n* = 1, multiple sclerosis *n* = 1). An additional 49 participants from the NCDS-Childhood study were not eligible for follow-up because they were deceased (*n* = 9), incarcerated or in probation (*n* = 12), or had moved away, were hospitalized, or could not be located (*n* = 28). Additionally, 28 adolescents declined to participate. The remaining 212 adolescents participated in the NCDS-Adolescent follow-up, including the present study. Inuit participants came from 14 Hudson Bay Coastal villages of Nunavik (Northern Quebec), around Puvirnituq (50%) Inukjuak (37%), and Kuujjuaraapik (13%). Participants from smaller villages in this region were transported by plane to meet with the research team.

### Ethical considerations

Interviews were conducted by a non-Inuit experienced research assistant in French or in English or by a native Inuit interpreter in Inuktituk. At the NCDS-Adolescent follow-up study, written informed consent was obtained from all participants aged 18 years and older. For participants under the age of 18, parental written informed consent was obtained together with the participant’s assent, as per ethical guidelines. The consent form stated that individual participant information would be kept confidential and anonymous by substituting codes for participant identifiers. Additionally, the form indicated that only anonymized data and study results would be disseminated and published. Data analysis and writing of the present study were conducted between January 2021 and September 2021. The research procedures were approved by Université Laval (Project #812-08-980-21) and CHU Sainte-Justine Research Centre (Project # 2934) ethics committees. This research was also approved by the Nunavik communities, the Nunavik Regional Board of Health and Social Services and of the Kativik Regional Government. Additional information regarding the ethical, cultural, and scientific considerations specific to inclusivity in global research is included in the Supporting information (S1 Checklist).

### Biospecimen collection and chemical analyses

Prenatal exposure to Pb, Hg and PCBs as well as nutrients, such as omega-3 fatty acids and selenium (Se), were obtained by collecting blood samples (30 mL) from the umbilical cord at birth [41, 46]. Child and adolescent exposures were obtained from venous blood sample at age 11 (20 mL) and 18 (30 mL), respectively. Pb levels were measured by graphite furnace atomic absorption spectroscopy with Zeeman background correction in umbilical cord blood samples and by inductively coupled plasma mass spectrometry (Perkin Elmer Sciex Elan 6000 ICP-MS instrument) in the child blood samples. Total (inorganic and organic) Hg concentrations were measured by cold vapor atomic absorption spectrometry in umbilical cord blood samples and by inductively coupled plasma mass spectrometry (PE DRC II instrument) in the child blood samples. Pb and Hg adolescent blood samples were both analyzed using inductively coupled plasma mass spectrometry (PerkinElmer ELAN ICP-MS DRC II). Concentrations of PCBs congeners (28, 52, 101, 105, 118, 128, 138, 153, 156, 170, 180, 183 and 187) were measured in blood plasma by dual capillary column high-resolution gas chromatography coupled with an electron capture detector. Because individual PCBs congeners are highly inter-correlated, only the most prevalent, non-dioxin-like congener 153, was used as a marker of total PCBs exposure [47]. Se concentration was obtained by using inductively coupled plasma mass spectrometry (ICP-MS). Cord and venous blood samples of environmental contaminants, selenium and total lipids were analyzed at the Centre de Toxicologie du Québec (Canada). Concentrations of docosahexaenoic acid (DHA) were expressed as percentage of the total area of all fatty acid peaks form C14:0 to C24:1 (percent weight), as described in Jacobson, Jacobson [44]. DHA cord and child samples were analyzed by the Lipid Analytical Laboratory at the University of Guelph, and DHA adolescent samples were analyzed at CHU de Québec.

### Sensation seeking assessment

Sensation seeking was assessed in all adolescent participants using two standardized self-report scales: the Brief Sensation Seeking Scale– 4 items scale (BSSS-4) [48], which targets general aspects of sensation seeking, and the Sensation Seeking– 2 items scale (SS-2) [49], which focuses on thrill and danger-related sensation seeking. We made sure that all participants were sufficiently fluent in English before administrating both questionnaires. The BSSS-4 [48] is a shortened version of the original Sensation Seeking Scale V (SSS-V) [50]. The BSSS-4 items represent a construct of sensation seeking with four factors (Cronbach α = 0.66) [48], namely, experience and novelty seeking (ENS, *I would like to explore strange places*), disinhibition (Dis, *I like to do frightening things*), thrill and adventure seeking (TAS, *I like new and exciting experiences*) and boredom susceptibility (BS, *I prefer friends who are exciting and unpredictable*) [26]. Each item is scored on a 5-point Likert scale ranging from “strongly disagree” to “strongly agree”, with a higher total score indicating greater sensation seeking. Although the BSSS-4 has not been validated in Inuit populations, it has been validated in various ethnic youth groups, including Caucasian, Hispanic and African American as well as Mexican, Peruvian and Italian youth [51–53]. The SS-2 is a 2-item questionnaire (Cronbach α = 0.83) [49], assessing thrill and danger seeking behaviors [48]. The two items, rated on a reversed 5-point Likert scale from “very often” to “not at all” are: (a) *How often do you do dangerous things for fun*? *and* (b) *How often do you do exciting things even if they are dangerous*? In comparison to more exhaustive sensation seeking questionnaires, this index was designed to assess excitement more directly in relation to social and physical risk-taking [54]. The SS-2 has previously been validated as a predictive measure of adolescent substance use behaviors [55] and violence-related media use [49].

### Potential confounding variables

A broad list of potential confounders was examined based on previous research. IQ was obtained from an estimated score of the Wechsler Intelligence Scale for Children (WISC-IV), i.e., adapted for use in the Inuit culture [43]. Principal provider’s SES was assessed at 11 and 18 years of age on the Hollingshead Index of Social Status, based on a weighted score of occupation and education level [56]. Participant’s household food security status was obtained using a modified version of the Household Food Security Survey Module [57]. As used in Bradette-Laplante, Courtemanche [58], 8 of the initial 18 items were used to collect food security data, such as whether the household could afford the food needed or whether members had to cut down the size of the meal. The final measure used was dichotomous for childhood food security status (secure vs insecure) and categorical for adolescent food security status (1 = food secure, 2 = food insecure without hunger, 3 = food insecure with hunger), based on Health Canada food security norms [59]. The following maternal characteristics were also included: age at delivery (years), whether the participants were breastfed (y/n), and whether the mother used tobacco (y/n), alcohol (y/n) and/or marijuana (y/n) during pregnancy. Nutrient concentrations, namely Se and DHA, were also examined as potential covariates, given their putative protective actions against environmental contaminants toxicity [60, 61]. Given the association between affective state and sensation seeking [37–39], affect was assessed using the Positive and Negative Affect Schedule (PANAS) [62], which consists of two 10-item self-report questionnaires that measure the extent to which an individual has experienced positive and negative affect over the last month (Cronbach α = 0.86 and 0.87) [62]. Each item corresponds to a single-word adjective (i.e., *Disgusted*, *Alert*, *Inspired*) and is rated on a 5-point Likert Scale from “very slightly or not at all” to “extremely”. On this scale, high positive affect refers to pleasurable engagement with the environment, reflected by positive emotions, such as interest and enthusiasm, while low positive affect refers to lack of positive emotions, namely, lethargy and sadness. High negative affect represents one’s unpleasurable engagement with the environment, reflected by aversive emotions, such as distress and nervousness, while low negative affect refers to the absence of such aversive states and represents states of calmness and tranquility [62, 63].

### Statistical analyses

The distribution of continuous variables was checked for normality. The total score on the BSSS-4 and SS-2 were normally distributed. Cord, child and adolescent Pb, Hg and PCB-153 concentrations as well as nutrient concentrations (i.e., Se and DHA) were log10-transformed because they were positively skewed. Maternal cannabis and alcohol use during pregnancy were missing for 13.21% of the cases (*n* = 28); cord blood selenium concentrations, for 8.02% (*n* = 17); and current food security status, for 5.19% (*n* = 11). We performed Little’s Test of Missing Completely at Random (MCAR) and found no indication of systematic bias arising from missingness (*X*^2^ = 168.60, df = 193, *p* = 0.90) [64]. Full information maximum likelihood (FIML) estimation was used in all analyses to minimize exclusion of participants due to missing data [65].

The associations between contaminants and self-report sensation seeking were assessed with a three-step hierarchical linear regression model. All dependent variables were Z-score standardized. In the first block, the exposure of interest (i.e., lead, mercury or PCB-153) for a given window of exposure (i.e., prenatal, child, or adolescent) was entered without adjustment. In the second block, positive and negative affect scores from the PANAS were entered as affective functioning known to be altered in chronically exposed individuals and those which may influence sensation seeking propensity. Age at assessment and sex of participant were systematically included in the third block, as well as total blood lipid concentrations in analyses in which PCB-153 was the independent variable. Additional covariates were included as confounders in the third block, if they were correlated with at least one contaminant and one sensation seeking total score at *p* ≤ 0.20. Regarding nutrients, blood nutrient levels from the same developmental period as the contaminant of interest were selected. Sensitivity analyses were performed to further adjust for simultaneous exposure to other contaminants by re-running the final model with adjustment for exposure to other toxicants during the same period of exposure (e.g., control for adolescent Pb and Hg exposure when assessing adolescent blood PCB-153 levels). To explore potential moderation of sex in the association between contaminant exposure and sensation seeking, secondary interaction analyses between sex and exposure were conducted. Interaction terms of sex by Pb, Hg or PCB-153 exposure were included in the final regression models. A statistical criterion of *p* ≤ 0.10) was used to justify follow-up sex-specific stratified regression analyses. All analyses were conducted with RStudio v. 1.4.1717. Results were considered statistically significant if *p* ≤ 0.05.

## Results

Descriptive characteristics of the study sample are presented in Table 1. Among the 212 participants, 207 had completed both BSSS-4 and SS-2, and no outliers (SD > 3) were detected. Age at completion of the sensation seeking questionnaires ranged between 16.01 and 21.88 years old (*M* = 18.47, SD = 1.15), and the sample consisted of a slightly higher proportion of females (55.67%). The vast majority of participants’ mothers (85.92%) reported smoking tobacco while pregnant. Males (*M* = 5.80, SD = 2.12) rated higher than females (*M* = 4.98, SD = 2.23) on the SS-2 questionnaire (*t* (205) = 2.69, *p* < 0.01), whereas no difference was found between males (*M* = 12.14, SD = 2.88) and females (*M* = 11.82, SD = 3.34) on the BSSS-4 questionnaire (*t* (205) = 0.72, *p* = 0.47). Considering that scores on the BSSS-4 (Cronbach α = 0.66) and the SS-2 (Cronbach α = 0.70) were weakly correlated (*r*_*s*_ = 0.27, *p* < 0.01), both outcomes were examined in separate analyses in the regression models. The mean and the variability of the scores as well as the internal consistency (Cronbach) for both the BSSS-4 and SS-2 scales were consistent with those observed in other adolescent studies [48, 49, 52, 53], thus supporting the validity of these questionnaires within our study. Intercorrelations between sensation seeking scores, PANAS scores and contaminants blood concentrations (log10) are shown in the supporting information (S1 Table).

**Table 1:** Descriptive characteristics of the study sample.

	*N*	%	Median	Mean	SD	Min-Max
Participant characteristics						
Age at assessment (yr)	212		18.48	18.47	1.15	16.01–21.88
Sex (% female)	212	55.67				
Estimated IQ at age 11 yr	212		90.50	92.38	11.58	70.00–125.00
Primary caregiver SES at 11-yr visit	212		28.25	28.59	11.38	8.00–66.00
Primary caregiver SES at 18-yr visit	210		21.00	21.58	9.03	11.00–47.00
Food security (% secure) at 11-yr visit	210	59.05				
Food security (% secure) at 18-yr visit	201	18.41				
Maternal characteristics						
Age at delivery (yr)	212		22.24	23.69	5.67	15.00–42.00
Breastfeeding (% yes)	207	74.88				
Alcohol use during pregnancy (% yes)	184	54.35				
Tobacco use during pregnancy (% yes)	206	85.92				
Marijuana use during pregnancy (% yes)	184	34.24				
Nutrient blood concentrations						
Cord Se (umol/L)	195		3.40	4.02	1.89	1.40–14.00
Cord DHA (% fatty acids)	212		3.50	3.60	1.27	1.12–7.73
Child Se (umol/L)	210		2.30	2.54	1.09	0.90–9.50
Child DHA (% fatty acids)	209		2.21	2.40	0.97	0.60–4.96
Adolescent Se (umol/L)	212		2.99	3.59	2.02	1.40–14.00
Adolescent DHA (% fatty acids)	212		3.32	3.35	1.21	0.60–7.12
Contaminant blood concentrations						
Cord Pb (umol/L)	204		0.18	0.22	0.15	0.04–0.86
Cord Hg (nmol/L)	204		76.50	100.13	79.14	9.00–495.00
Cord PCB-153 (ug/L)	205		0.22	0.30	0.28	0.04–2.42
Child Pb (umol/L)	210		0.10	0.13	0.11	0.03–0.62
Child Hg (nmol/L)	210		16.00	23.49	21.97	0.30–140.00
Child PCB-153 (ug/L)	209		0.23	0.38	0.43	0.02–3.40
Adolescent Pb (umol/L)	212		0.07	0.10	0.10	0.02–0.88
Adolescent Hg (nmol/L)	212		21.00	29.76	29.82	0.70–180.00
Adolescent PCB-153 (ug/L)	211		0.18	0.26	0.26	0.02–1.90
PANAS scores						
Negative affect	204		19.00	19.28	6.26	10.00–40.00
Positive affect	204		28.00	28.35	7.29	12.00–43.00
Sensation seeking scores						
BSSS-4	207		12.00	11.97	3.13	5.00–19.00
SS-2	207		5.00	5.36	2.21	1.00–10.00

SES: Socioeconomic status, IQ: Intelligence quotient, PANAS: Positive and Negative Affect Schedule, Se: Selenium, DHA: Docosahexaenoic acid, Pb: Lead, Hg: Mercury, PCBs: Polychlorinated biphenyls, BSSS-4: Brief Sensation Seeking Scale– 4, SS-2: Sensation Seeking– 2.

As shown in Table 2 (Model 1), results from unadjusted regression analyses indicated that child blood Pb levels were significantly associated with lower BSSS-4 total scores (b = -0.23, *p* = 0.001), while adolescent blood levels of Pb were only marginally linked to this outcome (b = -0.13, *p* = 0.08). Also, whereas child blood Hg levels fell just short of statistical significance (b = -0.11, *p* = 0.10), cord and adolescent blood Hg levels (b = -0.22, *p* = 0.001; b = -0.14, *p* = 0.04), were significantly associated with lower BSSS-4 total scores. As for PCB-153, blood concentrations at all three developmental time periods were significantly related to lower BSSS-4 total scores (b = -0.19, *p* = 0.007; b = -0.24, *p* = 0.001; b = -0.16, *p* = 0.02, respectively). Regarding risk-taking sensation seeking measured by the SS-2, no significant associations were found between Pb or Hg blood levels at any developmental period and SS-2 total scores. Cord and child blood PCB-153 levels were significantly associated with lower SS-2 total score (b = -0.18, *p* = 0.01; b = -0.16, *p* = 0.04), while adolescent blood PCB-153 levels were marginally linked to this outcome (b = -0.13, *p* = 0.06).

**Table 2:** Associations between cord, child and adolescent environmental contaminant exposures and sensation seeking scores during adolescence (*N* = 212).

	Model 1	Model 2	Model 3
	Unadjusted b (CI 95%)	Adjusted b (CI 95%)	Adjusted b (CI 95%)
BSSS-4 total score			
Cord			
Pb	-0.08 (-0.22, 0.05)	-0.09 (-0.22, 0.04)	-0.03 (-0.16, 0.10)
Hg	-0.22 (-0.36, -0.09)**	-0.17 (-0.30, -0.04)*	-0.10 (-0.25, 0.05)
PCB-153	-0.19 (-0.33, -0.05)**	-0.14 (-0.28, -0.001)*	-0.09 (-0.23, 0.04)
Child			
Pb	-0.23 (-0.36, -0.10)**	-0.19 (-0.32, -0.06)**	-0.18 (-0.32, -0.04)*
Hg	-0.11 (-0.25, 0.02)	-0.07 (-0.21, 0.06)	-0.04 (-0.20, 0.13)
PCB-153	-0.24 (-0.39, -0.10)**	-0.19 (-0.34, -0.05)**	-0.16 (-0.32, 0.004)†
Adolescent			
Pb	-0.13 (-0.26, 0.01)†	-0.10 (-0.23, 0.04)	-0.09 (-0.23, 0.05)
Hg	-0.14 (-0.27, -0.01)*	-0.12 (-0.25, 0.01)†	-0.17 (-0.33, -0.02)*
PCB-153	-0.16 (-0.30, -0.03)*	-0.12 (-0.25, 0.02)†	-0.10 (-0.26, 0.06)
SS-2 total score			
Cord			
Pb	-0.07 (-0.20, 0.07)	-0.07 (-0.20, 0.07)	-0.06 (-0.19, 0.08)
Hg	-0.02 (-0.15, 0.12)	0.02 (-0.12, 0.15)	0.02 (-0.14, 0.17)
PCB-153	-0.18 (-0.32, -0.04)*	-0.16 (-0.30, -0.01)*	-0.15 (-0.29, -0.01)*
Child			
Pb	-0.03 (-0.16, 0.11)	-0.004 (-0.14, 0.13)	-0.07 (-0.22, 0.07)
Hg	-0.05 (-0.19, 0.09)	-0.04 (-0.18, 0.10)	0.02 (-0.15, 0.19)
PCB-153	-0.16 (-0.31, -0.01)*	-0.14 (-0.29, 0.01)†	-0.12 (-0.29, 0.05)
Adolescent			
Pb	0.07 (-0.06, 0.21)	0.09 (-0.04, 0.23)	0.03 (-0.12, 0.17)
Hg	-0.03 (-0.17, 0.10)	-0.03 (-0.16, 0.11)	-0.09 (-0.26, 0.07)
PCB-153	-0.13 (-0.26, 0.01)†	-0.10 (-0.24, 0.03)	-0.24 (-0.39, -0.08)**

† *p* < 0.10.

* *p* < 0.05.

** *p* < 0.01

Model 1: unadjusted standardized regression coefficients.

Model 2: adjusted for PANAS positive and negative scores.

Model 3: adjusted for PANAS positive and negative scores and additional adjustment for sex, age at assessment, primary caregiver SES at testing time, IQ and food security status at age 11, breastfeeding status, prenatal tobacco exposure and concomitant selenium exposure.

To determine whether positive and negative affects act as confounders on the associations, regression models were adjusted by adding the PANAS positive and negative scores to the models (Table 2, Model 2). Significant changes defined by a Beta coefficient change of ≥ 10% were observed on the BSSS-4 when adjusting for positive and negative affect scores in all significant or marginally significant unadjusted models. PANAS scores also significantly influenced relations between the PCB-153 blood levels at all developmental periods and risk-taking sensation seeking scores measured by the SS-2. Of note, all associations remained in the same (negative) direction, so that more exposure was associated with lower sensation seeking. A further look at the individual PANAS regression coefficients revealed that self-report positive affect acted as a significant predictor of lower sensation seeking measured by the BSSS-4 in cord (b = 0.20, *p* = 0.01), child (b = -0.19, *p* = 0.01) and adolescent (b = -0.19, *p* = 0.01), such that higher positive affect scores were related to higher rates of global sensation seeking during adolescence. Negative affect did not significantly predict BSSS-4 total scores. For its part, lower risk-taking sensation seeking as assessed using the SS-2 was shown to be only marginally predicted by negative affect scores (b = -0.12, *p* = 0.08) in the adolescent model—higher rates of negative affect scores were related to higher rates of risk-taking sensation seeking. Positive affect scores did not act as a significant predictor of the SS-2 outcome.

The last of the regression models adjusting for all potential confounders are presented in Table 2 (Model 3). Results indicated that higher child blood levels of Pb (b = -0.18, *p* = 0.01) and PCB-153 (b = -0.16, *p* = 0.06) remained significantly associated with lower BSSS-4 total scores. The magnitude of these associations remained similar after controlling for exposure to other contaminants at the same time (S2 Table), i.e., child Hg and child PCB-153 for the child Pb model (b = -0.16, *p* = 0.03) and child Hg and child Pb for the child PCB-153 model (b = -0.15, *p* = 0.09). Adolescent blood levels for Hg were also associated with lower BSSS-4 total scores, when controlling for sociodemographic and nutrients confounders (b = -0.17, *p* = 0.03), but this association was no longer significant after adjustment for simultaneous exposure to other contaminants (S2 Table). Regarding risk-taking sensation seeking, after adjusting for relevant potential confounders, cord and adolescent blood PCB-153 levels remained significantly related to lower SS-2 total scores (b = -0.15, *p* = 0.04; b = -0.24, *p* = 0.004). The magnitude of the association remained similar in the cord PCB-153 model, after controlling for cord co-exposure to Pb and Hg (b = -0.16, *p* = 0.04), while the magnitude of the association increased in the adolescent PCB-153 model after control for adolescent co-exposures to Pb and Hg (b = -0.24, *p* = 0.005). Interestingly, whereas child PCB-153 blood levels were not significantly associated with SS-2 total scores after controlling for potential confounders (see Table 2, Model 3) (b = -0.12, *p* = 0.15), additional adjustment for Pb and Hg co-exposure during childhood showed a negative but marginal association between child PCB-153 blood levels and SS-2 total scores (b = -0.18, *p* = 0.05).

Results from secondary analyses regarding sex interactions are presented in S3 Table. The only sex interaction term to reach our statistical criterion (*p* < 0.10) was for child blood PCB-153 concentrations (b = -0.28, *p* = 0.05). Sex-stratified analyses showed that child blood PCB-153 concentrations were associated with lower SS-2 total score for the girls (b = -0.28, *p* = 0.01), whereas no significant associations were found for the boys (b = 0.11, *p* = 0.40).

## Discussion

The aim of the present study was to evaluate the associations between Pb, Hg and PCB developmental exposures and sensation seeking in adolescents from Northern Quebec (Nunavik). We expected the exposure levels and sensation seeking to be positively related. Contrary to our hypotheses, contaminant levels were, in general, associated with lower global sensation seeking. Associations of both child blood Pb and child blood PCB-153 levels with lower scores on the BSSS-4 scale remained significant after statistical adjustment for simultaneous exposure to other contaminants. Consistent with previous cohort studies showing detrimental effects of early childhood exposure to Pb on neurodevelopment [9, 10], we observed associations only in relation to child blood Pb concentrations, while none were found with either cord or concurrent (adolescent) blood levels. Furthermore, cord, child and adolescent PCB-153 concentrations were all linked to lower risk-related sensation seeking on the SS-2 scale, even after controlling for both Pb and Hg exposure. The latter findings raise the importance of examining postnatal exposures to toxic compounds together with prenatal exposures when assessing neurobehavioral outcomes in adolescence, as the brain is vulnerable to continuous changes throughout this critical developmental period.

Although our findings generally point towards negative associations between exposures and self-reported sensation seeking, there were some differences between results found on the BSSS-4 and the SS-2. Such discrepancies can be explained by the weak correlation that we found between the two questionnaires in our sample (*r*_*s*_ = 0.27), suggesting that they do not assess the same constructs. The BSSS-4 measures *general* sensation seeking, including novelty seeking, thrill and adventure seeking, disinhibition, boredom susceptibility; whereas the SS-2 more specifically assesses thrill and danger-related sensation seeking [48]. Therefore, the two questionnaires appear complementary, resulting in a broader assessment of sensation seeking constructs and a more detailed understanding of the associations being studied.

As shown in our study, PANAS scores exacerbated the relation between sensation seeking scores and contaminant exposures for several biomarkers (see Table 2, Model 2). These results suggest that sensation seeking acts as a function of contaminant blood levels, which are likely to be influenced by positive affect, and to a lesser extent, by negative affect, in Inuit adolescents. Various cross-sectional studies have shown associations between chronic low-level Pb exposure and mood problems, namely fatigue, anxiety and depressive symptoms in adult populations [11, 66]. Similarly, chronic exposure to Hg have been linked to depressive symptomatology in numerous cohorts [67–69]. Moreover, Plusquellec et al. [9] showed that Inuit preschoolers prenatally exposed to PCB-153 exhibited lower positive affect, according to examiner ratings of positive expressions (i.e., smiling, laughing). In addition, postnatal PCB exposure has been associated with higher rates of depressive symptoms in adults with occupational chronic exposure [12], while sensation seeking and reward approach behaviors have been correlated with higher rates of positive affect [70, 71]. By contrast, anhedonic and clinically-depressed individuals show less sensation seeking and lower motivation to incur risky decisions [72, 73]. In our sample, higher rates of negative affect (e.g., nervousness, distress) did not have significant effects on the relations between contaminant exposure and sensation seeking, whereas positive affect did influence associations of contaminant exposure to global sensation seeking. Given the negative correlations between contaminant exposure and positive affect at 18 years of age in our sample, our results suggest that developmental exposure to Pb, Hg and PCBs may be linked to lower positive interaction with one’s environment, including lethargy and sadness, which could, in turn, induce decreased motivation to seek pleasurable experiences and sensations in exposed adolescents. Additional, more in-depth studies are needed to assess the role of affect in the relations between contaminant exposure and sensation seeking tendencies in adolescent populations.

In our study sample, sexual dimorphism in sensation seeking was not clearly evident, although males rated higher than females on the risk-taking sensation seeking questionnaires, which is consistent with many studies showing less of a propensity for risk-taking in female adolescents [74, 75]. However, a sex-specific moderation of child blood PCB-153 levels was observed, with higher concentrations associated with lower risk-related sensation seeking in girls only. The fact that no other interaction effect was found between sex and exposures in relation to sensation seeking is somewhat surprising given, on the one hand, that PCBs can disrupt sex-specific hormonal activities [76–78], and, on the other, that sex-specific associations are often reported between legacy contaminants and neurobehavioral measures [79–81]. Although we cannot exclude the possibility of a spurious finding (only one significant interaction (p < 0.1) was found among all analyses), further studies are needed to replicate our moderation effect findings, not only for PCB but also for Pb and Hg.

To our knowledge, this is the first study to assess self-reported sensation seeking as a function of prenatal and postnatal exposure to legacy contaminants. Previous animal studies have assessed associations between chronic developmental exposures to Pb, Hg and PCBs and impulsivity extensively (see Perez-Fernandez, Flores [82] for review), a behavioral characteristic also linked to reward sensitivity and risk-taking behaviors [83]. In line with these studies, Stewart, Sargent [25] showed that in humans, prenatal exposure to Hg and PCBs as well as postnatal exposure to Pb were associated with deficits in impulse control in reaction to reinforcement. Furthermore, developmental exposure to heavy metals, principally Pb and PCBs, have been identified as risk factors for engagement in disinhibitory and externalizing behaviors (e.g., substance use, antisocial behaviors, hyperactivity) [84–86]. More recently, Vieira, Levy [87] assessed relations between cord blood Pb, Hg and PCBs levels and maladaptive behavioral regulation in adolescent risk-taking behaviors but failed to find any significant associations between these prenatal exposures and self-reported sensation seeking at age 15 years. This inconsistency with our findings underscores the importance of considering postnatal exposure in addition to prenatal exposure when assessing behaviors, such as propensity for sensation seeking, in exposed adolescence.

Our findings contribute to the current literature by showing that inverse relations between chronic exposure to legacy contaminants and sensation seeking could be due to other factors, including heightened impulsivity [9, 25] and cognitive deficits and/or emotional impairment [8, 88, 89]. Implications of high impulsivity combined with low sensation seeking in chronically-exposed individuals should be further examined in studies of general functioning and well-being as well as those involving risky behaviors occurring during adolescence. Although commonly associated with adverse outcomes, increased sensation seeking during adolescence can also promote the search for novel experiences from which individuals are likely to learn adaptative responses, acquire self-knowledge and develop self-sufficiency [90–92]. High levels of sensation seeking have also been associated with greater stress resiliency [93] and less physiological [94, 95] and behavioral [39, 96] reactivity to potential threats and aversive stimuli. Therefore, lower sensation seeking in Pb, Hg and PCBs chronically exposed Inuit adolescents can have potential repercussions on motivational and behavioral outcomes.

### Strengths and limitations

A strength of the study was the use of two distinct brief self-report questionnaires as measures of sensation seeking in adolescents, which improved our confidence in the observed associations. Moreover, the longitudinal design of the study enabled us to assess prenatal, childhood and current blood concentrations of environmental contaminants on sensation seeking using reliable and objective biomarkers of exposure, while taking into account numerous potential confounders from the prenatal period to late adolescence. Finally, we explored the role of emotional disturbances in the relation between contaminant exposure and behavioral features and demonstrated the need to account emotional factors when assessing associations between exposure to contaminants and reward-related behaviors.

Since some of our findings may be specific to the Inuit adolescent population from Nunavik, we should remain cautious regarding generalization of results to non-Inuit populations, given the unique sociocultural context of the Inuit population as well as their high levels of exposure to environmental contaminants compared to southern populations. In addition, neither the BSSS-4 nor the SS-2 questionnaires were previously validated in the Inuit populations. Therefore, there is a possibility that Inuit adolescents may express sensation seeking in a different way than that initially targeted by the questionnaire’s items. As with other epidemiological studies, it was not possible to consider all potential confounding variables. For instance, early life adversity (e.g., poverty, abuse, neglect) warrants consideration, given its link to lower sensation seeking in adolescents [97, 98] and that rates of early life adversity are elevated in Inuit population from Nunavik [99].

## Conclusion

The present study shows negative associations between developmental exposure to Pb, Hg and PCBs and self-reported sensation seeking in Inuit adolescents. Some of these associations were seemingly influenced by rates of positive affect in our participants. Sex differences observed in child blood PCB levels were associated with lower risk-taking sensation seeking in adolescent girls but not boys. Further research is needed to assess the extent to which lower sensation seeking in adolescents who are chronically exposed to environmental contaminants affects their behavior and well-being, paying particular attention to both emotional and neurochemical underlying mechanisms. More broadly, it is crucial to continue documenting effects of contaminant exposure on various neurobehavioral and neurocognitive features. By contributing to a better understanding of ways in which these contaminants impact neurodevelopment, we provide evidence-based data helping inform policy decision-makers. These data can also be used to promote reduction of contaminant production by large international industries, thereby helping to protect Inuit health and rights and support the continued wide-spread use of their highly nutritious country foods.

## Supporting information

S1 Table Spearman intercorrelations between sensation seeking scores, PANAS scores and contaminant blood concentrations (log10). (DOCX)

S2 Table Associations between cord, child and adolescent exposures and sensation seeking scores during adolescence after full adjustment (*N* = 212). (DOCX)

S3 Table Interaction terms of contaminant concentrations by sex on sensation seeking scores. (DOCX)

S1 Checklist Inclusivity in global research. (DOCX)

## References

[1] Muckle G, Ayotte P, Dewailly EE, Jacobson SW, Jacobson JL. Prenatal exposure of the northern Quebec Inuit infants to environmental contaminants. Environ Health Perspect. 2001;109(12):1291–9. Epub 2001/12/19. doi: 10.1289/ehp.011091291 ; PubMed Central PMCID: PMC1240513. 11748038 PMC1240513

[2] Barrie LA, Gregor D, Hargrave B, Lake R, Muir D, Shearer R, et al. Arctic contaminants: sources, occurrence and pathways. Sci Total Environ. 1992;122(1–2):1–74. Epub 1992/07/15. doi: 10.1016/0048-9697(92)90245-n . 1514103

[3] Adamou TY, Riva M, Muckle G, Laouan Sidi EA, Lemire M, Ayotte P. Blood mercury and plasma polychlorinated biphenyls concentrations in pregnant Inuit women from Nunavik: Temporal trends, 1992–2017. Sci Total Environ. 2020;743:140495. Epub 2020/08/08. doi: 10.1016/j.scitotenv.2020.140495 . 32758811

[4] Blanchet C, Rochette L. Nutrition and Food Consumption among the Inuit of Nunavik. Quebec, Canada: Institut national de Santé Publique du Québec & Nunavik Regional Board of Health and Social Services; 2008.

[5] Bélanger RE, Muckle G, Courtemanche Y, Poliakova N. Substance use ‐ Qanuilirpitaa? 2017 ‐ Nunavik Inuit Health Survey. Quebec, Canada: Nunavik Regional Board of Health and Social Services; 2020.

[6] Bélanger RE, Muckle G, Courtemanche Y, Poliakova N, Fletcher C, Lyonnais MC. Gambling, internet and media use ‐ Quanuilirpitaa? 2017 ‐ Nunavik Inuit Health Survey. Nunavik Regional Board of Health and Social Services; 2020.

[7] National Collaborating Centre for Aboriginal Health. The state of knowledge of aboriginal health: A review of aboriginal public health in Canada. British Colombia, Canada2012.

[8] Gagnon-Chauvin A, Bastien K, Saint-Amour D. Environmental toxic agents: The impact of heavy metals and organochlorides on brain development. Handb Clin Neurol. 2020;173:423–42. Epub 2020/09/23. doi: 10.1016/B978-0-444-64150-2.00030-7 . 32958188

[9] Plusquellec P, Muckle G, Dewailly E, Ayotte P, Begin G, Desrosiers C, et al. The relation of environmental contaminants exposure to behavioral indicators in Inuit preschoolers in Arctic Quebec. Neurotoxicology. 2010;31(1):17–25. Epub 2009/10/27. doi: 10.1016/j.neuro.2009.10.008 . 19854214

[10] Bellinger D, Leviton A, Allred E, Rabinowitz M. Pre- and postnatal lead exposure and behavior problems in school-aged children. Environ Res. 1994;66(1):12–30. Epub 1994/07/01. doi: 10.1006/enrs.1994.1041 . 8013435

[11] Bouchard MF, Bellinger DC, Weuve J, Matthews-Bellinger J, Gilman SE, Wright RO, et al. Blood lead levels and major depressive disorder, panic disorder, and generalized anxiety disorder in US young adults. Arch Gen Psychiatry. 2009;66(12):1313–9. Epub 2009/12/10. doi: 10.1001/archgenpsychiatry.2009.164 ; PubMed Central PMCID: PMC2917196. 19996036 PMC2917196

[12] Gaum PM, Esser A, Schettgen T, Gube M, Kraus T, Lang J. Prevalence and incidence rates of mental syndromes after occupational exposure to polychlorinated biphenyls. Int J Hyg Environ Health. 2014;217(7):765–74. Epub 2014/06/22. doi: 10.1016/j.ijheh.2014.04.001 . 24951400

[13] Lamoureux-Tremblay V, Muckle G, Maheu F, Jacobson SW, Jacobson JL, Ayotte P, et al. Risk factors associated with developing anxiety in Inuit adolescents from Nunavik. Neurotoxicol Teratol. 2020;81:106903. Epub 2020/06/09. doi: 10.1016/j.ntt.2020.106903 . 32512128 PMC7483563

[14] Zald DH, Treadway MT. Reward Processing, Neuroeconomics, and Psychopathology. Annu Rev Clin Psychol. 2017;13:471–95. Epub 2017/03/17. doi: 10.1146/annurev-clinpsy-032816-044957 ; PubMed Central PMCID: PMC5958615. 28301764 PMC5958615

[15] Schultz W. Dopamine signals for reward value and risk: basic and recent data. Behav Brain Funct. 2010;6:24. Epub 2010/04/27. doi: 10.1186/1744-9081-6-24 ; PubMed Central PMCID: PMC2876988. 20416052 PMC2876988

[16] Cory-Slechta DA, Pokora MJ, Preston RA. The effects of dopamine agonists on fixed interval schedule-controlled behavior are selectively altered by low-level lead exposure. Neurotoxicol Teratol. 1996;18(5):565–75. Epub 1996/09/01. doi: 10.1016/0892-0362(96)00082-7 . 8888021

[17] Rice DC. Lead exposure during different developmental periods produces different effects on FI performance in monkeys tested as juveniles and adults. Neurotoxicology. 1992;13(4):757–70. Epub 1992/01/01. . 1302302

[18] Brockel BJ, Cory-Slechta DA. Lead, attention, and impulsive behavior: changes in a fixed-ratio waiting-for-reward paradigm. Pharmacol Biochem Behav. 1998;60(2):545–52. Epub 1998/06/19. doi: 10.1016/s0091-3057(98)00023-9 . 9632239

[19] Paletz EM, Craig-Schmidt MC, Newland MC. Gestational exposure to methylmercury and n-3 fatty acids: effects on high- and low-rate operant behavior in adulthood. Neurotoxicol Teratol. 2006;28(1):59–73. Epub 2006/01/18. doi: 10.1016/j.ntt.2005.11.003 . 16413743

[20] Newland MC, Hoffman DJ, Heath JC, Donlin WD. Response inhibition is impaired by developmental methylmercury exposure: acquisition of low-rate lever-pressing. Behav Brain Res. 2013;253:196–205. Epub 2013/06/01. doi: 10.1016/j.bbr.2013.05.038 ; PubMed Central PMCID: PMC3832198. 23721962 PMC3832198

[21] Reed MN, Banna KM, Donlin WD, Newland MC. Effects of gestational exposure to methylmercury and dietary selenium on reinforcement efficacy in adulthood. Neurotoxicol Teratol. 2008;30(1):29–37. Epub 2007/12/22. doi: 10.1016/j.ntt.2007.10.003 ; PubMed Central PMCID: PMC2254940. 18096364 PMC2254940

[22] Rice DC. Effect of postnatal exposure to a PCB mixture in monkeys on multiple fixed interval-fixed ratio performance. Neurotoxicol Teratol. 1997;19(6):429–34. Epub 1997/12/11. doi: 10.1016/s0892-0362(97)87364-3 . 9392778

[23] Rice DC. Behavioral impairment produced by low-level postnatal PCB exposure in monkeys. Environ Res. 1999;80(2 Pt 2):S113–S21. Epub 1999/03/27. doi: 10.1006/enrs.1998.3917 . 10092425

[24] Sable HJ, Powers BE, Wang VC, Widholm JJ, Schantz SL. Alterations in DRH and DRL performance in rats developmentally exposed to an environmental PCB mixture. Neurotoxicol Teratol. 2006;28(5):548–56. Epub 2006/08/26. doi: 10.1016/j.ntt.2006.06.005 . 16930942

[25] Stewart PW, Sargent DM, Reihman J, Gump BB, Lonky E, Darvill T, et al. Response inhibition during Differential Reinforcement of Low Rates (DRL) schedules may be sensitive to low-level polychlorinated biphenyl, methylmercury, and lead exposure in children. Environ Health Perspect. 2006;114(12):1923–9. Epub 2006/12/23. doi: 10.1289/ehp.9216 ; PubMed Central PMCID: PMC1764150. 17185286 PMC1764150

[26] Zuckerman M. Behavioral expressions and biosocial bases of sensation seeking. New York: Cambridge University Press; 1994. 463 p.

[27] Quinn PD, Harden KP. Differential changes in impulsivity and sensation seeking and the escalation of substance use from adolescence to early adulthood. Dev Psychopathol. 2013;25(1):223–39. Epub 2012/07/25. doi: 10.1017/S0954579412000284 ; PubMed Central PMCID: PMC3967723. 22824055 PMC3967723

[28] Zheng Y, Tian M, Li Q, Liu X. Greater tolerance to losses in sensation seeking: Evidence from probability and delay discounting. Drug Alcohol Depend. 2019;194:159–65. Epub 2018/11/18. doi: 10.1016/j.drugalcdep.2018.09.027 . 30445273

[29] Bornovalova MA, Cashman-Rolls A, O’Donnell JM, Ettinger K, Richards JB, deWit H, et al. Risk taking differences on a behavioral task as a function of potential reward/loss magnitude and individual differences in impulsivity and sensation seeking. Pharmacol Biochem Behav. 2009;93(3):258–62. Epub 2008/12/02. doi: 10.1016/j.pbb.2008.10.023 . 19041886

[30] Desrichard O, Denarie V. Sensation seeking and negative affectivity as predictors of risky behaviors: a distinction between occasional versus frequent risk-taking. Addict Behav. 2005;30(7):1449–53. Epub 2005/07/19. doi: 10.1016/j.addbeh.2005.01.011 . 16022940

[31] Greene K, Krcmar M, Walters LH, Rubin DL, Jerold, Hale L. Targeting adolescent risk-taking behaviors: the contributions of egocentrism and sensation-seeking. J Adolesc. 2000;23(4):439–61. Epub 2000/08/11. doi: 10.1006/jado.2000.0330 . 10936016

[32] Steinberg L, Albert D, Cauffman E, Banich M, Graham S, Woolard J. Age differences in sensation seeking and impulsivity as indexed by behavior and self-report: evidence for a dual systems model. Dev Psychol. 2008;44(6):1764–78. Epub 2008/11/13. doi: 10.1037/a0012955 . 18999337

[33] Harden KP, Tucker-Drob EM. Individual differences in the development of sensation seeking and impulsivity during adolescence: further evidence for a dual systems model. Dev Psychol. 2011;47(3):739–46. Epub 2011/05/04. doi: 10.1037/a0023279 . 21534657

[34] Cross CP, Cyrenne DL, Brown GR. Sex differences in sensation-seeking: a meta-analysis. Sci Rep. 2013;3:2486. Epub 2013/08/31. doi: 10.1038/srep02486 ; PubMed Central PMCID: PMC3757272. 23989235 PMC3757272

[35] Spear LP. The adolescent brain and age-related behavioral manifestations. Neurosci Biobehav Rev. 2000;24(4):417–63. Epub 2000/05/19. doi: 10.1016/s0149-7634(00)00014-2 . 10817843

[36] Leather NC. Risk-taking behaviour in adolescence: a literature review. J Child Health Care. 2009;13(3):295–304. Epub 2009/08/29. doi: 10.1177/1367493509337443 . 19713410

[37] Littlefield AK, Stevens AK, Ellingson JM, King KM, Jackson KM. Changes in negative urgency, positive urgency, and sensation seeking across adolescence. Pers Individ Dif. 2016;90:332–7. Epub 2016/03/08. doi: 10.1016/j.paid.2015.11.024 ; PubMed Central PMCID: PMC4772734. 26949280 PMC4772734

[38] Carton S, Morand P, Bungenera C, Jouvent R. Sensation-seeking and emotional disturbances in depression: relationships and evolution. J Affect Disord. 1995;34(3):219–25. Epub 1995/06/08. doi: 10.1016/0165-0327(95)00020-n . 7560550

[39] Franken RE, Gibson KJ, Rowland GL. Sensation seeking and the tendency to view the world as threatening. Pers Individ Dif. 1991;13(1):31–8.

[40] Ernst M, Pine DS, Hardin M. Triadic model of the neurobiology of motivated behavior in adolescence. Psychol Med. 2006;36(3):299–312. Epub 2006/02/14. doi: 10.1017/S0033291705005891 ; PubMed Central PMCID: PMC2733162. 16472412 PMC2733162

[41] MuckleG, DewaillyE, AyotteP. Prenatal exposure of Canadian children to polychlorinated biphenyls and mercury. Can J Public Health. 1998;89 Suppl 1:S20–5, 2–7. Epub 1998/07/09. .9654788

[42] Dewailly E, Bruneau S, Ayotte P, Laliberté C, Gingras S, Bélanger D, et al. Health status at birth of inuit newborn prenatally exposed to organochlorines. Chemosphere. 1993;27(1–3):359–66.

[43] Jacobson JL, Muckle G, Ayotte P, Dewailly E, Jacobson SW. Relation of Prenatal Methylmercury Exposure from Environmental Sources to Childhood IQ. Environ Health Perspect. 2015;123(8):827–33. Epub 2015/03/11. doi: 10.1289/ehp.1408554 ; PubMed Central PMCID: PMC4529008. 25757069 PMC4529008

[44] Jacobson JL, Jacobson SW, Muckle G, Kaplan-Estrin M, Ayotte P, Dewailly E. Beneficial effects of a polyunsaturated fatty acid on infant development: evidence from the inuit of arctic Quebec. J Pediatr. 2008;152(3):356–64. Epub 2008/02/19. doi: 10.1016/j.jpeds.2007.07.008 . 18280840

[45] Dallaire R, Dewailly E, Ayotte P, Forget-Dubois N, Jacobson SW, Jacobson JL, et al. Growth in Inuit children exposed to polychlorinated biphenyls and lead during fetal development and childhood. Environ Res. 2014;134:17–23. Epub 2014/07/22. doi: 10.1016/j.envres.2014.06.023 ; PubMed Central PMCID: PMC4262554. 25042032 PMC4262554

[46] Dewailly EE, Ayotte P, Bruneau S, Laliberte C, Muir DC, Norstrom RJ. Inuit exposure to organochlorine through the aquatic food chain in arctic québec. Environ Health Perspect. 1993;101(7):618–20. doi: 10.1289/ehp.93101618 8143594 PMC1519892

[47] Ayotte P, Muckle G, Jacobson JL, Jacobson SW, Dewailly E, Inuit Cohort S. Assessment of pre- and postnatal exposure to polychlorinated biphenyls: lessons from the Inuit Cohort Study. Environ Health Perspect. 2003;111(9):1253–8. Epub 2003/07/05. doi: 10.1289/ehp.6054 ; PubMed Central PMCID: PMC1241583. 12842782 PMC1241583

[48] Stephenson MT, Hoyle RH, Palmgreen P, Slater MD. Brief measures of sensation seeking for screening and large-scale surveys. Drug Alcohol Depend. 2003;72(3):279–86. Epub 2003/12/04. doi: 10.1016/j.drugalcdep.2003.08.003 . 14643945

[49] Slater MD. Alienation, aggression, and sensation-seeking as predictors of adolescent use of violent film, computer and website content. J Commun. 2003;53:105–21.

[50] Zuckerman M, Eysenck S, Eysenck HJ. Sensation seeking in England and America: cross-cultural, age, and sex comparisons. J Consult Clin Psychol. 1978;46(1):139–49. Epub 1978/02/01. doi: 10.1037//0022-006x.46.1.139 PubMed PMID: 627648. 627648

[51] Vallone D, Allen JA, Clayton RR, Xiao H. How reliable and valid is the Brief Sensation Seeking Scale (BSSS-4) for youth of various racial/ethnic groups? Addiction. 2007;102 Suppl 2:71–8. Epub 2007/10/06. doi: 10.1111/j.1360-0443.2007.01957.x . 17850616

[52] Merino-Soto C, Salas Blas E. Brief Sensation Seeking Scale: Latent structure of 8-item and 4-item versions in Peruvian adolescents. Adicciones. 2018;30(1):41–53. Epub 2017/05/12. doi: 10.20882/adicciones.842 . 28492952

[53] Merino-Soto C, Salas-Blas E, Perez-Amezcua B, Garcia-Rivas J, Pena OIG, Toledano-Toledano F. Brief Sensations Seeking Scale (BSSS): Validity Evidence in Mexican Adolescents. Int J Environ Res Public Health. 2022;19(13). Epub 2022/07/10. doi: 10.3390/ijerph19137978 ; PubMed Central PMCID: PMC9265267. 35805633 PMC9265267

[54] Slater MD. Sensation-seeking as a moderator of the effects of peer influences, consistency with personal aspirations, and perceived harm on marijuana and cigarette use among younger adolescents. Subst Use Misuse. 2003;38(7):865–80. Epub 2003/06/13. doi: 10.1081/ja-120017614 . 12801146

[55] Slater MD, Hoyle R, Stephenson MT, Palmgreen P. A reliable two-item sensation-seeking index and prediction of substance use. Society for Prevention Research; Washington, DC2001.

[56] Hollingshead AB. Four factor index of social status. Yale J Sociol. 2011;8:21–51.

[57] Bickel G, Nord M, Price C, Hamilton W, Cook J. Guide to measuring household food security. Revised. 2000.

[58] Bradette-Laplante M, Courtemanche Y, Desrochers-Couture M, Forget-Dubois N, Belanger RE, Ayotte P, et al. Food insecurity and psychological distress in Inuit adolescents of Nunavik. Public Health Nutr. 2020;23(14):2615–25. Epub 2020/05/28. doi: 10.1017/S1368980020000117 . 32456742 PMC10200505

[59] Office of Nutrition Policy and Promotion Health Products and Food Brand. Canadian Community Health Survey, Cycle 2.2, Nurition (2004): income-related household food security in Canada. Ottawa, Ontario: Health Canada; 2007.

[60] Choi AL, Budtz-Jorgensen E, Jorgensen PJ, Steuerwald U, Debes F, Weihe P, et al. Selenium as a potential protective factor against mercury developmental neurotoxicity. Environ Res. 2008;107(1):45–52. Epub 2007/09/15. doi: 10.1016/j.envres.2007.07.006 ; PubMed Central PMCID: PMC2538682. 17854796 PMC2538682

[61] Skoczynska A, Wojakowska A, Nowacki D, Bobak L, Turczyn B, Smyk B, et al. Unsaturated fatty acids supplementation reduces blood lead level in rats. Biomed Res Int. 2015;2015:189190. Epub 2015/06/16. doi: 10.1155/2015/189190 ; PubMed Central PMCID: PMC4446462. 26075218 PMC4446462

[62] Watson D, Clark LA, Tellegen A. Development and validation of brief measures of positive and negative affect: the PANAS scales. J Pers Soc Psychol. 1988;54(6):1063–70. Epub 1988/06/01. doi: 10.1037//0022-3514.54.6.1063 . 3397865

[63] Crawford JR, Henry JD. The positive and negative affect schedule (PANAS): construct validity, measurement properties and normative data in a large non-clinical sample. Br J Clin Psychol. 2004;43(Pt 3):245–65. Epub 2004/08/31. doi: 10.1348/0144665031752934 . 15333231

[64] Little RJA. A Test of Missing Completely at Random for Multivariate Data with Missing Values. Journal of the American Statistical Association. 1988;83(404):1198–202. doi: 10.1080/01621459.1988.10478722

[65] Enders CK, Bandalos DL. The relative performance of full information maximum likelihood estimation for missing data in structural equation models. Struct Equ Model A Multidiscip J. 2001;8(3):430–57. doi: 10.1207/S15328007SEM0803_5

[66] Rhodes D, Spiro A, 3rd, Aro A, Hu H. Relationship of bone and blood lead levels to psychiatric symptoms: the normative aging study. J Occup Environ Med. 2003;45(11):1144–51. Epub 2003/11/12. doi: 10.1097/01.jom.0000094995.23808.7b . 14610395

[67] Kim KW, Sreeja SR, Kwon M, Yu YL, Kim MK. Association of Blood Mercury Level with the Risk of Depression According to Fish Intake Level in the General Korean Population: Findings from the Korean National Health and Nutrition Examination Survey (KNHANES) 2008–2013. Nutrients. 2020;12(1). Epub 2020/01/16. doi: 10.3390/nu12010189 ; PubMed Central PMCID: PMC7019861. 31936641 PMC7019861

[68] Bagheri Hosseinabadi M, Khanjani N, Mobarake MD, Shirkhanloo H. Neuropsychological effects of long-term occupational exposure to mercury among chloralkali workers. Work. 2020;66(3):491–8. Epub 2020/07/12. doi: 10.3233/WOR-203194 . 32651342

[69] Yorifuji T, Tsuda T, Inoue S, Takao S, Harada M. Long-term exposure to methylmercury and psychiatric symptoms in residents of Minamata, Japan. Environ Int. 2011;37(5):907–13. Epub 2011/04/08. doi: 10.1016/j.envint.2011.03.008 . 21470684

[70] Herman AM, Critchley HD, Duka T. Risk-Taking and Impulsivity: The Role of Mood States and Interoception. Front Psychol. 2018;9:1625. Epub 2018/09/14. doi: 10.3389/fpsyg.2018.01625 ; PubMed Central PMCID: PMC6123387. 30210421 PMC6123387

[71] Merchan-Clavellino A, Alameda-Bailen JR, Zayas Garcia A, Guil R. Mediating Effect of Trait Emotional Intelligence Between the Behavioral Activation System (BAS)/Behavioral Inhibition System (BIS) and Positive and Negative Affect. Front Psychol. 2019;10:424. Epub 2019/03/21. doi: 10.3389/fpsyg.2019.00424 ; PubMed Central PMCID: PMC6411706. 30890980 PMC6411706

[72] Padrao G, Mallorqui A, Cucurell D, Marco-Pallares J, Rodriguez-Fornells A. Neurophysiological differences in reward processing in anhedonics. Cogn Affect Behav Neurosci. 2013;13(1):102–15. Epub 2012/09/13. doi: 10.3758/s13415-012-0119-5 . 22968926

[73] Carton S, Bungener C, Montreuil M, Allilaire JF, Widlocher D, Jouvent R. [Sensation seeking and mood dimensions in depressive states]. Encephale. 1992;18(5):567–74. Epub 1992/09/01. . 1340805

[74] Byrnes JP, Miller DC, Schafer WD. Gender differences in risk taking: A meta-analysis. Psychological Bulletin. 1999;125(3):367–83.

[75] Shulman EP, Harden KP, Chein JM, Steinberg L. Sex differences in the developmental trajectories of impulse control and sensation-seeking from early adolescence to early adulthood. J Youth Adolesc. 2015;44(1):1–17. Epub 2014/04/01. doi: 10.1007/s10964-014-0116-9 . 24682958

[76] Goncharov A, Rej R, Negoita S, Schymura M, Santiago-Rivera A, Morse G, et al. Lower serum testosterone associated with elevated polychlorinated biphenyl concentrations in Native American men. Environ Health Perspect. 2009;117(9):1454–60. Epub 2009/09/15. doi: 10.1289/ehp.0800134 ; PubMed Central PMCID: PMC2737025. 19750113 PMC2737025

[77] Fossato da Silva DA, Teixeira CT, Scarano WR, Favareto AP, Fernandez CD, Grotto D, et al. Effects of methylmercury on male reproductive functions in Wistar rats. Reprod Toxicol. 2011;31(4):431–9. Epub 2011/01/26. doi: 10.1016/j.reprotox.2011.01.002 . 21262343

[78] Ronis MJ, Gandy J, Badger T. Endocrine mechanisms underlying reproductive toxicity in the developing rat chronically exposed to dietary lead. J Toxicol Environ Health A. 1998;54(2):77–99. Epub 1998/07/04. doi: 10.1080/009841098158935 . 9652546

[79] Sagiv SK, Thurston SW, Bellinger DC, Altshul LM, Korrick SA. Neuropsychological measures of attention and impulse control among 8-year-old children exposed prenatally to organochlorines. Environ Health Perspect. 2012;120(6):904–9. Epub 2012/02/24. doi: 10.1289/ehp.1104372 ; PubMed Central PMCID: PMC3385436. 22357172 PMC3385436

[80] Cauli O, Piedrafita B, Llansola M, Felipo V. Gender differential effects of developmental exposure to methyl-mercury, polychlorinated biphenyls 126 or 153, or its combinations on motor activity and coordination. Toxicology. 2013;311(1–2):61–8. Epub 2012/12/12. doi: 10.1016/j.tox.2012.11.016 . 23220684

[81] Ris MD, Dietrich KN, Succop PA, Berger OG, Bornschein RL. Early exposure to lead and neuropsychological outcome in adolescence. J Int Neuropsychol Soc. 2004;10(2):261–70. Epub 2004/03/12. doi: 10.1017/S1355617704102154 . 15012846

[82] Perez-Fernandez C, Flores P, Sanchez-Santed F. A Systematic Review on the Influences of Neurotoxicological Xenobiotic Compounds on Inhibitory Control. Front Behav Neurosci. 2019;13:139. Epub 2019/07/25. doi: 10.3389/fnbeh.2019.00139 ; PubMed Central PMCID: PMC6620897. 31333425 PMC6620897

[83] Martin LE, Potts GF. Reward sensitivity in impulsivity. Neuroreport. 2004;15(9):1519–22. Epub 2004/06/15. doi: 10.1097/01.wnr.0000132920.12990.b9 . 15194887

[84] Dickerson AS, Ransome Y, Karlsson O. Human prenatal exposure to polychlorinated biphenyls (PCBs) and risk behaviors in adolescence. Environ Int. 2019;129:247–55. Epub 2019/05/31. doi: 10.1016/j.envint.2019.04.051 ; PubMed Central PMCID: PMC6605040. 31146159 PMC6605040

[85] Boucher O, Burden MJ, Muckle G, Saint-Amour D, Ayotte P, Dewailly E, et al. Response inhibition and error monitoring during a visual go/no-go task in inuit children exposed to lead, polychlorinated biphenyls, and methylmercury. Environ Health Perspect. 2012;120(4):608–15. Epub 2011/12/07. doi: 10.1289/ehp.1103828 ; PubMed Central PMCID: PMC3339450. 22142904 PMC3339450

[86] Desrochers-Couture M, Courtemanche Y, Forget-Dubois N, Belanger RE, Boucher O, Ayotte P, et al. Association between early lead exposure and externalizing behaviors in adolescence: A developmental cascade. Environ Res. 2019;178:108679. Epub 2019/08/28. doi: 10.1016/j.envres.2019.108679 ; PubMed Central PMCID: PMC6759380. 31454729 PMC6759380

[87] Vieira VM, Levy JI, Fabian MP, Korrick S. Assessing the relation of chemical and non-chemical stressors with risk-taking related behavior and adaptive individual attributes among adolescents living near the New Bedford Harbor Superfund site. Environ Int. 2021;146:106199. Epub 2020/10/31. doi: 10.1016/j.envint.2020.106199 ; PubMed Central PMCID: PMC7775916. 33126063 PMC7775916

[88] Gump BB, Dykas MJ, MacKenzie JA, Dumas AK, Hruska B, Ewart CK, et al. Background lead and mercury exposures: Psychological and behavioral problems in children. Environ Res. 2017;158:576–82. Epub 2017/07/18. doi: 10.1016/j.envres.2017.06.033 ; PubMed Central PMCID: PMC5562507. 28715786 PMC5562507

[89] Fruh V, Rifas-Shiman SL, Amarasiriwardena C, Cardenas A, Bellinger DC, Wise LA, et al. Prenatal lead exposure and childhood executive function and behavioral difficulties in project viva. Neurotoxicology. 2019;75:105–15. Epub 2019/09/13. doi: 10.1016/j.neuro.2019.09.006 ; PubMed Central PMCID: PMC6842061. 31513824 PMC6842061

[90] Ellis BJ, Del Giudice M, Dishion TJ, Figueredo AJ, Gray P, Griskevicius V, et al. The evolutionary basis of risky adolescent behavior: implications for science, policy, and practice. Dev Psychol. 2012;48(3):598–623. Epub 2011/11/30. doi: 10.1037/a0026220 . 22122473

[91] Norbury A, Husain M. Sensation-seeking: Dopaminergic modulation and risk for psychopathology. Behav Brain Res. 2015;288:79–93. Epub 2015/04/25. doi: 10.1016/j.bbr.2015.04.015 . 25907745

[92] Yoneda T, Ames ME, Leadbeater BJ. Is there a positive side to sensation seeking? Trajectories of sensation seeking and impulsivity may have unique outcomes in young adulthood. J Adolesc. 2019;73:42–52. Epub 2019/04/13. doi: 10.1016/j.adolescence.2019.03.009 . 30978586

[93] Norbury A, Kurth-Nelson Z, Winston JS, Roiser JP, Husain M. Dopamine Regulates Approach-Avoidance in Human Sensation-Seeking. Int J Neuropsychopharmacol. 2015;18(10):pyv041. Epub 2015/04/11. doi: 10.1093/ijnp/pyv041 ; PubMed Central PMCID: PMC4648156. 25857822 PMC4648156

[94] Lissek S, Powers AS. Sensation seeking and startle modulation by physically threatening images. Biol Psychol. 2003;63(2):179–97. Epub 2003/05/10. doi: 10.1016/s0301-0511(03)00053-x . 12738407

[95] De Pascalis V, Valerio E, Santoro M, Cacace I. Neuroticism-Anxiety, Impulsive-Sensation Seeking and autonomic responses to somatosensory stimuli. Int J Psychophysiol. 2007;63(1):16–24. Epub 2006/08/11. doi: 10.1016/j.ijpsycho.2006.06.004 . 16899317

[96] Blankstein KR. The sensation seeker and anxiety reactivity: relationships between the sensation-seeking scales and the activity preference questionnaire. J Clin Psychol. 1975;31(4):677–81. Epub 1975/10/01. . 1194425

[97] Loman MM, Johnson AE, Quevedo K, Lafavor TL, Gunnar MR. Risk-taking and sensation-seeking propensity in postinstitutionalized early adolescents. J Child Psychol Psychiatry. 2014;55(10):1145–52. Epub 2014/02/21. doi: 10.1111/jcpp.12208 ; PubMed Central PMCID: PMC4138294. 24552550 PMC4138294

[98] Kopetz C, Woerner JI, MacPherson L, Lejuez CW, Nelson CA, Zeanah CH, et al. Early psychosocial deprivation and adolescent risk-taking: The role of motivation and executive control. J Exp Psychol Gen. 2019;148(2):388–99. Epub 2018/09/18. doi: 10.1037/xge0000486 ; PubMed Central PMCID: PMC7181402. 30221961 PMC7181402

[99] Fortin S, Jacobson SW, Gagnon J, Forget-Dubois N, Dionne G, Jacobson JL, et al. Socioeconomic and psychosocial adversity in Inuit mothers from Nunavik during the first postpartum year. Journal of Aboriginal Health. 2015:63–75.

