# Supplementary material for: Pre- and postnatal exposure to legacy environmental contaminants and sensation seeking in Inuit adolescents from Nunavik
Source: PLOS Glob Public Health. 2023 Oct 18;3(10):e0002478. doi: 10.1371/journal.pgph.0002478 (PMC10584110; doi:10.1371/journal.pgph.0002478)
Supplement: S1 Table — (DOCX) [file pgph.0002478.s001.docx]

S1 Table. Spearman intercorrelations between sensation seeking scores, PANAS scores and contaminant blood concentrations (log10).

|  |  | Cord | | | Child | | | Adolescent | | | PANAS | | SS | |
| --- | --- | --- | --- | --- | --- | --- | --- | --- | --- | --- | --- | --- | --- | --- |
|  |  | Pb | Hg | PCB-153 | Pb | Hg | PCB-153 | Pb | Hg | PCB-153 | Negative | Positive | BSSS | SS2 |
| Cord | Pb | 1 |  |  |  |  |  |  |  |  |  |  |  |  |
|  | Hg | .15* | 1 |  |  |  |  |  |  |  |  |  |  |  |
|  | PCB-153 | .23* | .31** | 1 |  |  |  |  |  |  |  |  |  |  |
| Child | Pb | .17* | .11 | .07 | 1 |  |  |  |  |  |  |  |  |  |
|  | Hg | .14* | .42** | .19** | .23** | 1 |  |  |  |  |  |  |  |  |
|  | PCB-153 | .21** | .31** | .37** | .27** | .53** | 1 |  |  |  |  |  |  |  |
| Adolescent | Pb | .28** | .16* | .09 | .48** | .18** | .20** | 1 |  |  |  |  |  |  |
|  | Hg | .09 | .32** | .25** | .13† | .53** | .41** | .22** | 1 |  |  |  |  |  |
|  | PCB-153 | .13 | .37** | .41** | .31** | .56** | .82** | .25** | .49** | 1 |  |  |  |  |
| PANAS | Negative | .04 | -.08 | -.07 | -.09 | .03 | -.1 | -.08 | .03 | -.13 | 1 |  |  |  |
|  | Positive | .32** | .05 | -.21** | -.18* | -.13† | -.18* | -.17* | -.09 | -.09 | -.15* | 1 |  |  |
| SS | BSSS-4 | -.09 | -.23** | -.19** | -.23** | -.10 | -.21** | -.14* | -.11 | -.16* | -.13† | .31** | 1 |  |
|  | SS-2 | -.09 | -.03 | -.16* | -.02 | -.05 | -.18* | .10 | -.04 | -.11 | .15* | .15* | .27** | 1 |

† *p* < 0.10. * *p* < 0.05. ** *p* < 0.01

Pb: Lead, Hg: Mercury, PCB: Polychlorinated biphenyl, PANAS: Positive and Negative Schedule, SS: Sensation seeking, BSSS-4: Brief Sensation Seeking Scale – 4, SS2: Sensation Seeking – 2.
