# Supplementary material for: Pre- and postnatal exposure to legacy environmental contaminants and sensation seeking in Inuit adolescents from Nunavik
Source: PLOS Glob Public Health. 2023 Oct 18;3(10):e0002478. doi: 10.1371/journal.pgph.0002478 (PMC10584110; doi:10.1371/journal.pgph.0002478)
Supplement: S2 Table — (DOCX) [file pgph.0002478.s002.docx]

S2 Table. Associations between cord, child and adolescent exposures and sensation seeking scores during adolescence after full adjustment (*N* = 212)

|  | Adjusted β (CI 95%) |
| --- | --- |
| BSSS-4 total score |  |
| Cord |  |
| Pb | -0.01 (-0.14, 0.13) |
| Hg | -0.08 (-0.24, 0.07) |
| PCB-153 | -0.07 (-0.22, 0.07) |
| Child |  |
| Pb | -0.16 (-0.30, -0.02)* |
| Hg | 0.04 (-0.13, 0.22) |
| PCB-153 | -0.15 (-0.32, 0.02)† |
| Adolescent |  |
| Pb | -0.05 (-0.20, 0.09) |
| Hg | -0.14 (-0.31, 0.04) |
| PCB-153 | -0.05 (-0.22, 0.12) |
| SS-2 total score |  |
| Cord levels |  |
| Pb | -0.04 (-0.17, 0.10) |
| Hg | 0.06 (-0.10, 0.22) |
| PCB-153 | -0.16 (-0.30, -0.01)* |
| Child (log) |  |
| Pb | -0.06 (-0.20, 0.09) |
| Hg | 0.09 (-0.09, 0.27) |
| PCB-153 | -0.17, (-0.35, 0.003)† |
| Adolescent |  |
| Pb | 0.06 (-0.08, 0.21) |
| Hg | -0.01 (-0.19, 0.17) |
| PCB-153 | -0.24 (-0.41, -0.07)** |

† *p* < 0.10. * *p* < 0.05. ** *p* < 0.01

β = standardized regression coefficients.

Models were adjusted for sex, age at assessment, PANAS positive and negative scores, primary caregiver SES at testing time, IQ and food security status at age 11 years, breastfeeding status, prenatal tobacco exposure, concomitant selenium exposure with additional adjustments for simultaneous exposure to other contaminants (Hg, Pb and/or PCB-153).
