# Supplementary material for: Pre- and postnatal exposure to legacy environmental contaminants and sensation seeking in Inuit adolescents from Nunavik
Source: PLOS Glob Public Health. 2023 Oct 18;3(10):e0002478. doi: 10.1371/journal.pgph.0002478 (PMC10584110; doi:10.1371/journal.pgph.0002478)
Supplement: S3 Table — (DOCX) [file pgph.0002478.s003.docx]

S3 Table. Interaction terms of contaminant concentrations by sex on

sensation seeking scores

|  | Adjusted β (CI 95%) | *p* value |
| --- | --- | --- |
| BSSS-4 total score |  |  |
| Cord |  |  |
| Pb x sex | 0.06 (-0.35, 0.47) | 0.79 |
| Hg x sex | 0.23 (-0.51, 0.98) | 0.54 |
| PCB-153 x sex | 0.05 (-0.26, 0.35) | 0.76 |
| Child |  |  |
| Pb x sex | 0.06 (-0.14, 0.26) | 0.56 |
| Hg x sex | 0.23 (-0.19, 0.64) | 0.29 |
| PCB-153 x sex | -0.09 (-0.37, 0.19) | 0.52 |
| Adolescent |  |  |
| Pb x sex | 0.37 (-0.20, 0.94) | 0.21 |
| Hg x sex | 0.12 (-0.29, 0.54) | 0.56 |
| PCB-153 x sex | 0.04 (-0.29, 0.37) | 0.81 |
| SS-2 total score |  |  |
| Cord |  |  |
| Pb x sex | -0.15 (-0.57, 0.28) | 0.50 |
| Hg x sex | 0.07 (-0.71, 0.85) | 0.87 |
| PCB-153 x sex | -0.13 (-0.45, 0.18) | 0.41 |
| Child |  |  |
| Pb x sex | -0.02 (-0.23, 0.20) | 0.88 |
| Hg x sex | -0.15 (-0.57, 0.28) | 0.51 |
| PCB-153 x sex | -0.28 (-0.56, 0.004) | 0.05† |
| Adolescent |  |  |
| Pb x sex | 0.07 (-0.52, 0.66) | 0.81 |
| Hg x sex | 0.03 (-0.40, 0.46) | 0.88 |
| PCB-153 x sex | -0.12 (-0.45, 0.21) | 0.48 |

†: *p* < 0.10.

β = standardized regression coefficients.

Models were adjusted for sex, age at assessment, PANAS positive and negative scores, primary caregiver SES at time of testing, IQ and food security status at age 11 years, breastfeeding status, prenatal tobacco exposure and concomitant selenium exposure.
