# Supplementary material for: Pre- and postnatal exposure to legacy environmental contaminants and sensation seeking in Inuit adolescents from Nunavik
Source: PLOS Glob Public Health. 2023 Oct 18;3(10):e0002478. doi: 10.1371/journal.pgph.0002478 (PMC10584110; doi:10.1371/journal.pgph.0002478)
Supplement: S1 Checklist — (DOCX) [file pgph.0002478.s004.docx]

Inclusivity in global research

PLOS’ policy on inclusivity in global research aims to improve transparency in the reporting of research performed outside of researchers’ own country or community and ensures that PLOS publications reporting global research adhere to high standards for research ethics and authorship. Authors of relevant research articles may be asked to complete the questionnaire below, which outlines ethical, cultural, and scientific considerations specific to inclusivity in global research. This questionnaire may be requested when researchers have travelled to a different country to conduct research, if research uses samples collected in another country, research with Indigenous populations or their lands, or if research is on cultural artefacts. Researchers travelling to another country solely to use laboratory equipment will not normally be required to complete the questionnaire. However, the questionnaire can be requested at the journal’s discretion for any submission – if you have been requested to complete this questionnaire by the PLOS journal you submitted to, please do so.

Please complete the questionnaire below and include this as a Supporting Information file with your manuscript. Note that if your paper is accepted for publication, this checklist will be published with your article in the supporting information files. Please ensure that you reference the checklist in the main body of your manuscript. We suggest adding a subsection ‘Inclusivity in global research’ to your Methods section and adding the following sentence: “Additional information regarding the ethical, cultural, and scientific considerations specific to inclusivity in global research is included in the Supporting Information (SX Checklist)”

The questions have been designed to be applicable to a wide range of study types, and there are subsections for both human subjects research and non-human subjects research. If any of the questions are not relevant to your research please mark them as “N/A” as appropriate.

**Ethical considerations, permits and authorship**

*This section is applicable to all research types.*

Provide details as to who granted permissions and/or consent for the study to take place in the Methods section of your manuscript. This should include the names of **all** ethics boards, governmental organizations, community leaders or other bodies that provided approval for the study. If individuals provided approval refer to these people by their role or title but do not list their name(s).

Reported on page number: 10

If there were any deviations from the study protocol after approval was obtained please provide details of these changes in the Methods section of your manuscript.
Did this study involve local collaborators that are residents of the country where the research was conducted or members of the community studied? If you do not have any authors from said communities, please provide an explanation for this below.

Reported on page number: N/A

Our research group has a strong partnership with the Nunavik Nutrition and Health Committee (NNHC), a committee which plays a crucial role in promoting collaboration, addressing regional concerns and monitoring ethical standards in research about health, nutrition, and contaminants in Nunavik. Every study from our research group underwent a review process to ensure NNHC’s approval and to take into account the community's interests. This collaborative approach ensures that our study was conducted with respect for and input from the community studied, despite the absence of authors from said community. The NNHC is composed of both Inuit and non-Inuit members, is under the leadership of the Director of Public Helath of the region, and Inuit members are appointed by major public organizations, including the regional government; these organizations are led by Boards of Directors composed exclusively of Inuit.

The NNHC is considered by Ethical Review Boards (ERB) of Universities and Research Centers of the province of Quebec as the reference committee responsible for authorizing health research involving Nunavik Inuit. The ERB have requested that we obtain the official support from the NNHC before they provide the ethic authorization.

Everyone listed as an author should meet PLOS’ criteria for authorship and all individuals who meet these criteria should be included in the author byline, rather than the acknowledgements. Authorship criteria is based on the International Committee of Medical Journal Editors (ICMJE) Uniform Requirements for Manuscripts Submitted to Biomedical Journals - for further information please see here: <https://journals.plos.org/plosone/s/authorship>.

**Human subjects research (e.g. health research, medical research, cross-cultural psychology)**

Did you obtain written informed consent from a representative of the local community or region before the research took place? How did you establish who speaks for the community? Details of written informed consent obtained from study participants should be reported separately in the Methods section of your manuscript.

Our study is aligned with the concerns of the Nunavik Regional Board of Health and Social Services and the Nunavik Nutrition and Health Committee (NNHC), with whom we have a close research relationship, about the impact of exposure to ubiquitous environmental contaminants on health within Indigenous communities. For more than 20 years, we have provided the NNHC and the Public Health of Nunavik the detailed findings from our research to advance better understanding of this issue and how the research can help them. Thus, we approached our study with the intention of contributing to a better understanding of the potential effects of environmental contaminants on adolescent behavior and well-being within the context of Inuit communities, as requested by the NNHC.

Details of written informed consent obtained from study participants are reported in the Methods section of our manuscript.

How did members of the local community provide input on the aims of the research investigation, its methodology, and its anticipated outcome(s)?

During the development of the cohort study, we met with the municipal councils of several communities, the management of the two hospitals and the Nunavik Regional Board of Health and Social Services to seek their support for the project. They suggested that we include certain themes so that the data generated would also address their concerns (e.g., food insecurity, substance dependence and pregnancy, etc.) Throughout the longitudinal study, our research team has maintained ongoing communication and collaboration with these groups. The results of each collection phase were the subject of a communication campaign under the leadership of the NNHC and the Public Health Department. This partnership allowed us to gain valuable insights and perspectives, ensuring that our research aligns with the community's priorities and needs.

When engaging with the local community, how did you ensure that the informed consent documents and other materials could be understood by local stakeholders?

The NNHC is composed of both Inuit and non-Inuit members. The communication language of the NNHC meetings are all in English, including the ones in which we presented our research projects. This allows an effective and comprehensive exchange of ideas and information, ensuring that our research objectives, methodology, and outcomes are clearly understood by all members of the NNHC. Moreover, when the NNHC recommended that we engage with local communities for dissemination of specific study results, written materials were provided in French, English and Inuktitut, which subsequently went through a review process by NNHC members and the Public Health director, so the documents were endorsed by them and were disseminated with their help.

Will the findings of the research be made available in an understandable format to stakeholders in the community where the study was conducted (e.g. via a presentation, summary report, copies of publications, etc.)? Please provide details of how this will be achieved.

Before submitting any article to a scientific journal, we always present the research findings to the NNHC members. As such, we provide them with the final draft of our manuscript and a lay summary that explains the study's context, methods, results, and conclusions in an accessible language. Our team is available to answer their questions or provide further explanations regarding the article's content. The NNHC members provide feedback that is integrated into the manuscript prior to submission to a scientific journal. The NNHC determines if generated results need to be made available and how. This process involves the research team, the Public Health Director and the NNHC. The NNHC and the Director of Public Health did not believe that the results of this specific publication needed to be disseminated to the communities, since they corroborate those already disseminated and for which Public Health recommendations related to consumption of country food were already provided.

**Non-human subjects research using specimens/ animals collected as part of the study, or those housed in archival collections. Examples include archaeology, paleontology, botany and zoology.**

Did the permission you obtained from a local authority to perform the study include an agreement on access to outputs and benefit sharing? This may include procedures to enable fair distribution of the benefits and resources arising from the research performed. Please include any details of Prior Informed Consent and Benefit Sharing Agreements obtained. These may be required by field-specific regulations, for example the Convention on Biological Diversity (CBD) and the associated Nagoya Protocol.

N/A

If the material used in your study was imported, please A) provide the year it was imported and B) indicate whether permits were obtained to import/export the materials used, C) provide details of any permits obtained. If this information is not available, please indicate this.

N/A

If you used archival specimens, please state how the material used in your study was acquired by the institute it is held in and provide details of any permits obtained for the original excavations/ sample collection. If this information is not available, please indicate this.

N/A

How was the potential cultural significance of the materials collected in your study to local communities considered in your research design? Were Indigenous peoples and/or local researchers and institutions involved with archaeological excavations / collection of specimens? If so, please provide a description of their involvement.

N/A

If your manuscript includes photographs of human remains please indicate whether authors obtained permission from descendants or affiliated cultural communities to do so.

N/A
